# Diagnostic performance of contrast-enhanced T2-FLAIR MRI in the detection of meningitis

**DOI:** 10.4102/sajr.v29i1.3018

**Published:** 2025-01-27

**Authors:** P. Sanjay, Vittal Manohar, Sushmita Balol, Yashwanth M.B. Naik

**Affiliations:** 1 Department of Radiodiagnosis, Mysore Medical College and Research Institute, Mysore, India

**Keywords:** meningitis, meninges, abnormal meningeal enhancement, contrast-enhanced T2-FLAIR, contrast-enhanced T1W, magnetic resonance imaging, single pixel signal intensity, central nervous system, retrospective study

## Abstract

**Background:**

The contrast-enhanced T2-FLAIR (CE-T2-FLAIR) sequence on MRI, through the suppression of CSF and vascular signals, can detect subtle meningeal enhancement in meningitis that may not be appreciable on the routinely used contrast-enhanced T1W (CE-T1W) sequence.

**Objectives:**

To assess CE-T2-FLAIR compared to CE-T1W in the diagnosis of meningitis, using CSF analysis as the gold standard, using both qualitative and quantitative approaches for assessment.

**Method:**

A retrospective study was conducted on 53 patients with clinically suspected meningitis referred for brain MRI. Twenty-seven patients, positive for meningitis on CSF analysis, were classified as the case group; the remaining patients were designated as controls. The pre-contrast, CE-T1W and CE-T2-FLAIR images were assessed and analysed, qualitatively for the detection of abnormal meningeal enhancement, and quantitatively by measuring single pixel signal intensities (SPSI) over the meninges and vessels.

**Results:**

Contrast-enhanced T2-FLAIR demonstrated significantly higher sensitivity (92.59% vs. 57.69%), negative predictive value (92.59% vs. 70.27%) and diagnostic accuracy (94.34% vs. 78.85%) compared to CE-T1W. Additionally, CE-T2-FLAIR showed significantly greater meningeal SPSI and enhancement than CE-T1W.

**Conclusion:**

Contrast-enhanced T2-FLAIR is better at detecting abnormal meningeal enhancement in meningitis than CE-T1W, because of significantly greater signal intensity and enhancement of the meninges compared to vessels.

**Contribution:**

This study reiterates the usefulness of CE-T2-FLAIR as an additional sequence for the detection of abnormal meningeal enhancement in cases of meningitis as confirmed both qualitatively and quantitatively.

## Introduction

Meningitis is a disease characterised by inflammation of the meninges.^1^ The meninges consist of three layers: the inner pia mater, which lines the brain and spinal cord, the middle arachnoid mater and the outer dura mater which lines the inner surface of the skull and forms dural folds such as the falx cerebri, tentorium and falx cerebelli.^2,3,4^ Meningitis can be caused by viral, bacterial, fungal, tubercular or parasitic infections.^5^ Viral meningitis is more common than bacterial meningitis, but bacterial meningitis is associated with a worse prognosis.

Meningitis is a major global health issue with 2.51 million cases reported in 2019; the age-standardised incidence ranges from 29.6 to 42.5 per 100 000 cases (mean ~35.4 per 100 000 cases) and the mortality rate ranges from 2.8 to 3.9 (mean~ 3.3) per 100 000 cases globally. The incidence and mortality rate are much higher in the under-5 age group. Apart from the high mortality rates, it can also be associated with high morbidity, because of cognitive impairment, neurological deficits, epilepsy and other complications.^1^

Clinical features of meningitis include fever, headache, altered sensorium and neck rigidity. Meningitis is characterised by elevated CSF opening pressure and leukocyte count.^2^ Analysis of CSF is the gold standard for diagnosing meningitis; however, it requires a lumbar puncture, an invasive procedure that poses risks in the presence of raised intracranial pressure and is associated with complications such as CSF leak. Cerebrospinal fluid studies may be non-contributary or culture results may be delayed.^6^ The risk of mortality from meningitis may increase 8.4-fold with a > 6 h delay in antibiotic therapy following presentation.^7^ Imaging such as MRI plays a vital role in the detection of meningitis and its complications.

Meningeal thickening that occurs in meningitis can be detected in only a few cases on pre-contrast MRI sequences. Abnormal meningeal enhancement (AME) is an important feature in detecting meningitis^6,8^ and must be differentiated from normal meningeal enhancement. Normal meningeal enhancement is subtle, thin and discontinuous, while AME is usually asymmetrical, thick, irregular, nodular, long (> 3 cm) segment, noted on more than three contiguous sections or extends into the sulcal bases.^3,5,6,9^ Contrast-enhanced T1-weighted (CE-T1W) imaging after the intravenous administration of gadolinium-based contrast agents is routinely used to detect meningeal or parenchymal enhancement. The contrast agents act by shortening the T1 relaxation time of protons. Other post-contrast sequences that can be used are proton-density, T2 Fluid attenuated inversion recovery (T2-FLAIR) and T1-weighted (T1W) sequence with magnetisation transfer (MT).^5,6^ Slow-flowing vessels in sulcal spaces might appear mildly hyperintense on T1W, which may be difficult to differentiate from meningeal enhancement. T2-FLAIR imaging suppresses signals from slow-flowing vessels because of the lack of inflow enhancement.^6^ The suppression of CSF signal, lack of vascular enhancement as compared to T1W imaging and the T1 relaxivity effect make it easier to appreciate meningeal enhancement on contrast-enhanced T2-FLAIR (CE-T2-FLAIR) compared to CE-T1W. Continuous enhancement on more than three sections is not required on CE-T2-FLAIR to consider AME. However, the use of the CE-T2-FLAIR sequence is limited by CSF pulsation artefact hyperintensity in the posterior fossa and lower relaxivity.^6,10^

Many studies have documented the superiority of CE-T2-FLAIR over CE-T1W imaging in the detection of meningitis.^6,8,10,11,12,13^ While most studies have qualitatively assessed meningeal enhancement, according to our knowledge only two studies have quantitatively assessed the meningeal enhancement.^6,10^ The aim of this study was to qualitatively and quantitatively assess the usefulness of CE-T2-FLAIR in meningitis compared to CE-T1W.

## Research methods and design

This retrospective study included all patients with clinically suspected meningitis who were referred for brain MRI to the Department of Radiodiagnosis at Mysore Medical College and Research Institute, over a 12-month period from 10 August 2023 to 09 August 2024. Patients who were positive for meningitis on CSF analysis were classified into the case group while those with negative results were placed in the control group. The CSF analysis results were obtained from hospital records.

**FIGURE 1:**
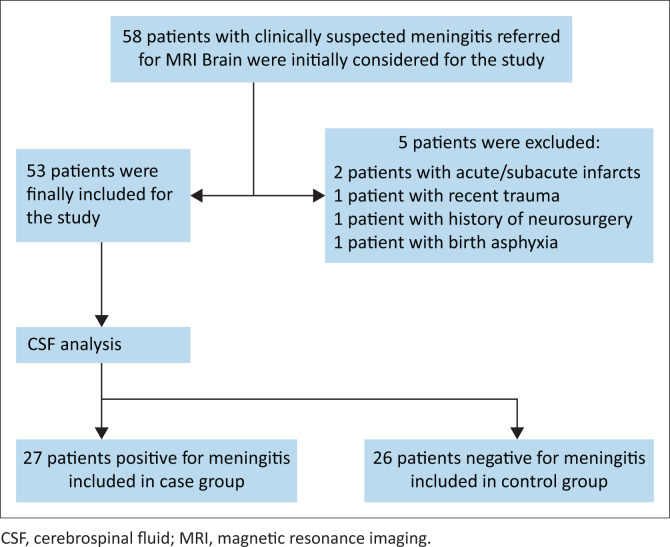
Flowchart of patient selection.

Any patients for whom CE-T2-FLAIR and CE-T1W were not performed, or for whom CSF analysis results were unavailable, were excluded from the study. Additionally, patients with contraindications to MRI, allergies to gadolinium contrast agents, impaired renal function, recent trauma, neurosurgery, brain and spine neoplasms, acute or subacute infarcts, congenital or birth abnormalities of the brain, or those who received supplemental oxygen, intravenous gadolinium or iodinated contrast in the previous week were also excluded. The study selection flow chart is presented in Figure 1.

### MRI protocol

Imaging was performed using a 1.5 Tesla (United Imaging, Jiading, Shanghai, China) uMR570 machine. All patients or guardians provided consent for brain MRI with contrast. The T1W sequence protocol was: time to repeat (TR): 16.77, time to echo (TE): 7.7, Slice thickness: 1, Slice gap: 1, Echo train length: 130, Echo number: 1, FOV: 232 × 256, TI:1100, Flip angle: 10, Acquisition matrix: 256 × 232, Pixel bandwidth: 160, Number of signal averages: 1 and Acquisition time: 3 min 54 s. Water excitation method of fat suppression was used. The T2-FLAIR sequence protocol was: TR: 6000, TE: 372, Slice gap: 0.7, Slice thickness: 0.7, Echo train length: 200, FOV: 232 × 256, TI: 1797, Flip angle: 54, Acquisition Matrix: 240 × 218, Pixel bandwidth: 500, Number of signal averages: 1 and Acquisition time: 4 min 5 s. Pre-contrast T1W and T2-FLAIR sequences were obtained. Contrast-enhanced T1-weighted and CE-T2-FLAIR sequences were obtained after the intravenous administration of gadolinium contrast agent (0.1 mmol/kg body weight of Gadobutrol). Contrast-enhanced T2-FLAIR was acquired after the CE-T1W sequence with a delay of nearly 4 min – 5 min.

### Image analysis

The MR images were retrieved from the picture archiving and communication system (PACS) and were evaluated randomly by two radiologists, who were blinded to the CSF analysis results. The pre-contrast and post-contrast T1W and T2-FLAIR sequences were evaluated to detect any meningeal abnormality. Readers were free to adjust window settings and scroll through the entire stack of MRI data.

#### Qualitative assessment

The exam was considered positive if the radiologist detected any AME. On CE-T1W, AME was considered definite if it was thick, long or noted on greater than three contiguous images. It was deemed equivocal if seen on less than three contiguous images or if it was not separately distinguishable from vascular enhancement. On CE-T2-FLAIR, any enhancement along the subarachnoid spaces or cranial nerves was considered AME. Leptomeningeal enhancement was graded as follows: grade 0 (no enhancement), grade 1 (thin meningeal enhancement, along < 3 sulcal spaces, but no enhancement along the basal cisterns), grade 2 (thick meningeal enhancement, along >3 sulcal spaces, but no enhancement along the basal cisterns), and grade 3 (enhancement along the basal cisterns ± cortical sulci).^6,10^ Image quality and diagnostic confidence were evaluated individually by using a five-point Likert scale (1, unacceptable; 5, excellent).

#### Quantitative assessment

In cases with AME, single pixel signal intensities (SPSI) were obtained from the regions of interest (ROI), of size 1 mm^2^, in the areas of meningeal and vascular enhancement, by placing a cursor at nearly the same position, on both the pre-contrast and post-contrast sequences. The difference in the SPSI in the meninges and vessels between pre-contrast and post-contrast sequences was used to calculate meningeal and vascular enhancement, respectively. An average of two measurements was taken for both readers. The average vascular enhancement was then subtracted from the average meningeal enhancement to obtain the net enhancement.^6,10^

### Data analysis

The data collected were recorded and entered into the Microsoft Excel (Microsoft, Redmond, Washington, United States) master sheet. Data were tabulated and analysed using the Statistical Package for Social Sciences (SPSS) version 24 software (IBM Corporation, Armonk, New York, United States). Categorical data were presented as numbers and percentages. Qualitative variables were analysed using Pearson’s Chi-square test and Fisher exact tests. Quantitative variables were presented using means and standard deviations (s.d.) and analysed using the student *t* test. A *p*-value < 0.05 was considered statistically significant. Sensitivity, specificity, positive predictive values (PPV), negative predictive values (NPV) and accuracy were calculated. All values of overall sensitivity, specificity, PPV, NPV and accuracy were expressed as means with 95% confidence intervals. For qualitative assessment, an inter-reader agreement was evaluated by computing weighted Cohen’s κ (kappa). Kappa results were qualitatively stratified by score κ = 0.81 to 1.00, almost perfect agreement; κ = 0.61 to 0.80, substantial agreement; κ = 0.41 to 0.60, moderate agreement; κ = 0.21 to 0.40, fair agreement; and κ < 0.20, slight agreement.^14^ For quantitative assessment, inter-reader agreement was evaluated by the Intraclass Correlation Coefficient (ICC). Intraclass Correlation Coefficient values < 0.5 indicate poor agreement, 0.5 to < 0.75 indicate moderate agreement, 0.75 to < 0.9 indicate good agreement and 0.90 to 1.0 indicate excellent agreement.^15^

### Ethical considerations

This retrospective study was approved by the Institutional Ethics Committee of Mysore Medical College and Research Institute and Associated Hospitals, Mysore. Ethical committee clearance was obtained on 17 August 2024. Patient consent for individual cases was waived, as all data were retrospectively collected from the institutional PACS and anonymised prior to review by individual readers.

## Results

A final total of 53 patients (29 females and 24 males), with clinically suspected meningitis, were included in the study. Twenty-seven patients who were CSF positive for meningitis were included in the case group; the rest of the patients were included in the control group. Table 1 includes the age and sex distribution in the sample.

**TABLE 1:** Distribution of age and sex in case and control groups.

Group	Total (*n*)	Sex		Age (years)		
		Male (*n*)	Female (*n*)	Mean	s.d.	Range
Case group	27	14	13	23.56	18.99	18 days to 65 years
Control group	26	10	16	28.95	25.57	22 days to 75 years
Case + control group	53	24	29	26.20	22.40	18 days to 75 years

*n*, number of patients; s.d., standard deviation.

### Qualitative assessment

**FIGURE 2:**
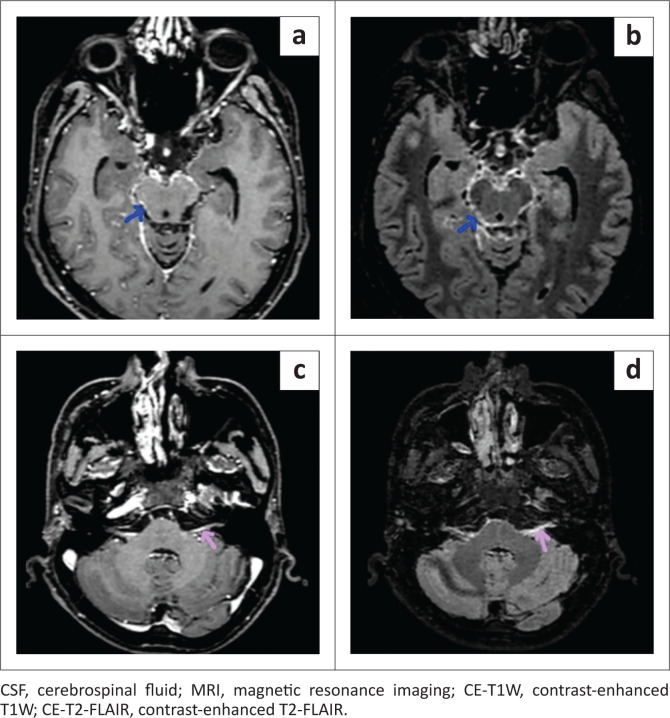
Axial brain MRI of a 30-year-old male patient with CSF proven meningitis. Basal cisternal enhancement (blue arrow) and cranial nerve enhancement (pink arrow) are more prominently seen on CE-T2-FLAIR (b and d) than on CE-T1W (a and c).

**FIGURE 3:**
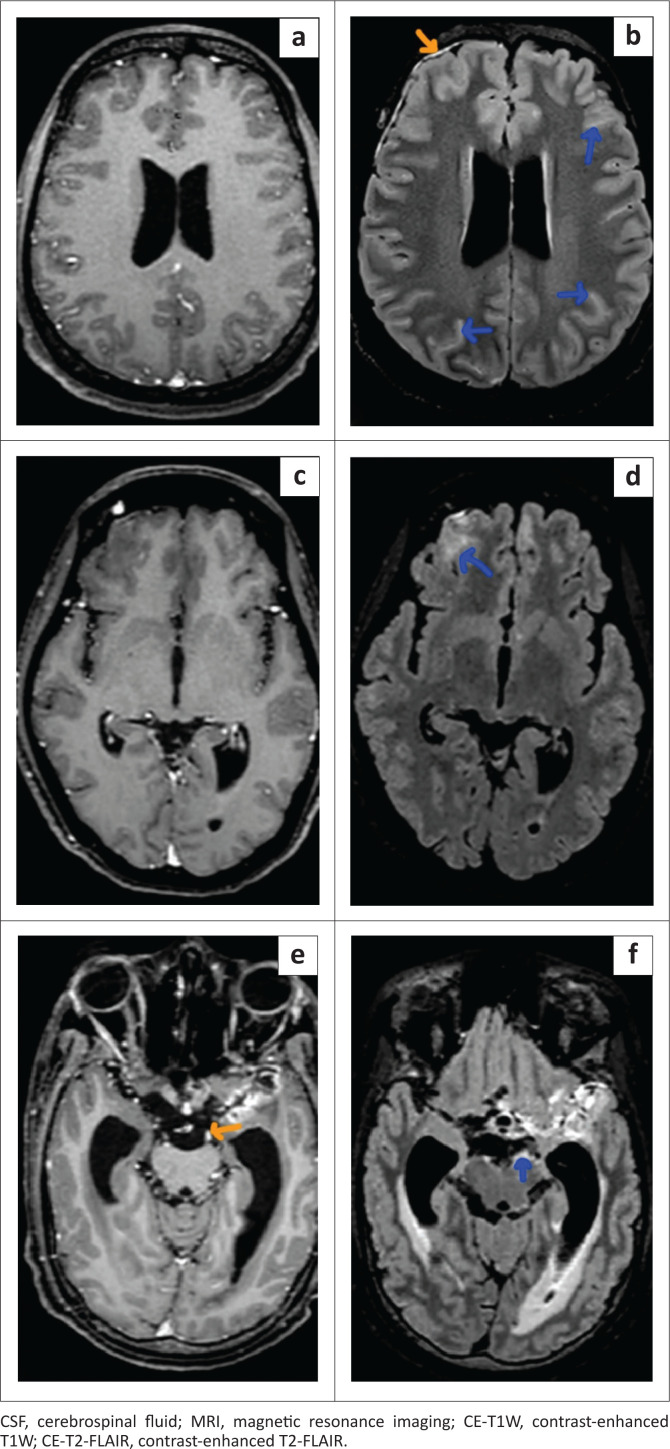
Axial brain MRI of three different patients. (a and b) A 36-year-old male patient with CSF proven meningitis. Thin sulcal enhancement along both cerebral hemispheres (blue arrows) and pachymeningeal enhancement along the right frontal lobe (orange arrow) are seen on the CE-T2-FLAIR (b) and not appreciated on CE-T1W (a). (c and d) A 40-year-old female with CSF proven meningitis. Thin sulcal enhancement along the right frontal lobe (blue arrow) is seen on CE-T2-FLAIR (d) and not appreciated on CE-T1W (c). (e and f) A 35-year-old male patient with CSF proven meningitis. Meningeal enhancement around the midbrain is seen on CE-T2-FLAIR (f) (blue arrow) and not seen in CE-T1W (e) (orange arrow represents a vessel).

**FIGURE 4:**
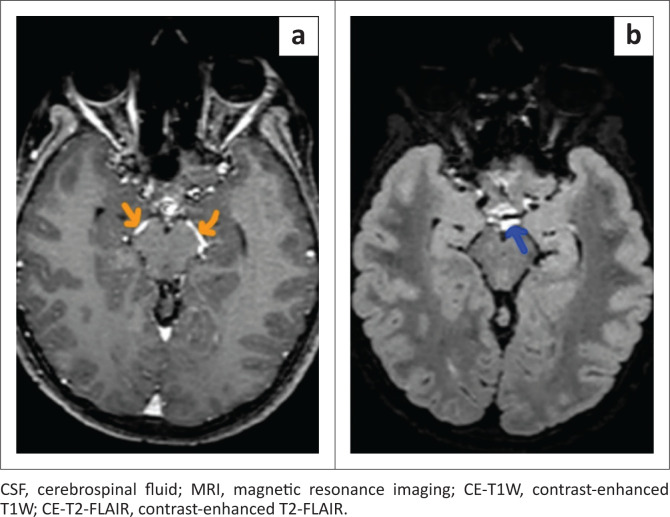
Axial brain MRI of a 15-year-old female negative for meningitis on CSF analysis. (a) CE-T1W shows enhancing vessels (orange arrows) around the midbrain. (b) CE-T2-FLAIR shows false positive basal cisternal enhancement (blue arrow) anterior to the midbrain.

**FIGURE 5:**
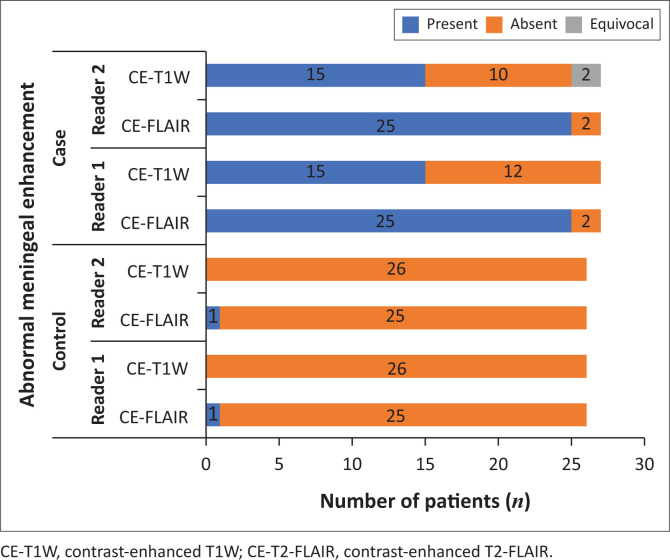
Bar chart showing a number of patients with abnormal meningeal enhancement in the case and control groups.

There was a significant difference in the qualitative assessment of meningeal abnormalities between CE-T1W and CE-T2-FLAIR (Figure 2, Figure 3 and Figure 4). In the case group, AME was found in a higher number of patients on CE-T2-FLAIR (25/27) than on CE-T1W (15/27). In the control group, there was one false positive AME on CE-T2-FLAIR, whereas no false positive AME was noted on CE-T1W. Reader 2 noted equivocal enhancement in two patients of the case group on CE-T1W (Figure 5).

**FIGURE 6:**
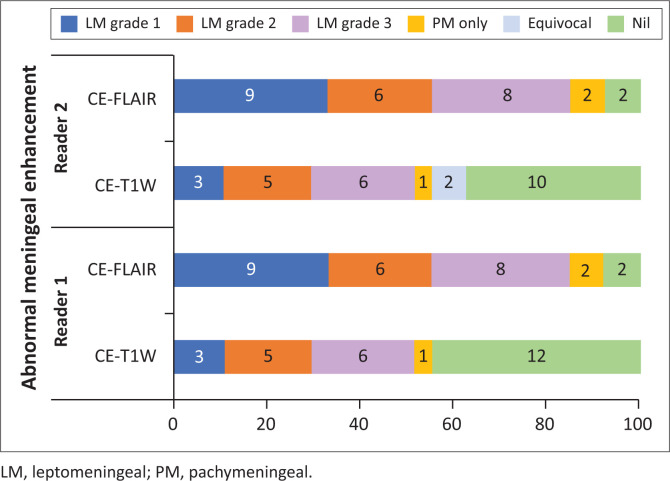
Bar chart showing the distribution of type and grade of abnormal meningeal enhancement in the case group.

Contrast-enhanced T2-FLAIR had a significantly higher sensitivity (92.59% vs. 57.69%) (*p* < 0.001), NPV (92.59% vs. 70.27%) (*p* < 0.001) and diagnostic accuracy (94.34% vs. 78.85%) compared to CE-T1W (*p* = 0.002) (combined reader 1 and reader 2). Contrast-enhanced T2-FLAIR and CE-T1W showed no significant difference in specificity (96.15% vs. 100%) (*p* = 0.298) and PPV (96.15% vs. 100%) (*p* = 0.374) (Table 2). The number of cases of AME as detected by each of the readers in CE-T1W and CE-T2-FLAIR have been represented in the bar graph (Figure 6).

**TABLE 2:** Comparison of contrast-enhanced T1W and contrast-enhanced T2-FLAIR in qualitative assessment of meningitis.

Sequence	Sensitivity		Specificity		PPV		NPV		Diagnostic accuracy	
	%	95% CI	%	95% CI	%	95% CI	%	95% CI	%	95% CI
**Reader 1**										
CE-T1W	55.56	35.33–74.52	100.00	86.77–100.00	100.00	78.20–100.00	68.42	58.70–76.76	77.36	63.79–87.72
CE-T2-FLAIR	92.59	75.71–99.09	96.15	80.36–99.90	96.15	78.48–99.42	92.59	76.67–97.94	94.34	84.34–98.82
***p***	< 0.01	-	0.522	-	0.617	-	< 0.001	-	< 0.05	-
**Reader 2**										
CE-T1W	60.00	38.67–78.87	100.00	86.77–100.00	100.00	78.20–100.00	72.22	61.67–80.78	80.39	66.88–90.18
CE-T2-FLAIR	92.59	75.71–99.09	96.15	80.36–99.90	96.15	78.48–99.42	92.59	76.67–97.94	94.34	84.34–98.82
***p***	< 0.01	-	0.523	-	0.617	-	< 0.01	-	< 0.05	-
**Combined reader 1 and reader 2**										
CE-T1W	57.69	43.20–71.27	100	93.15–100.00	100.00	88.43–100.00	70.27	63.25–76.45	78.85	69.74–86.24
CE-T2-FLAIR	92.59	82.11–97.94	96.15	86.79–99.53	96.15	86.50–98.98	92.59	82.94–96.98	94.34	88.09–97.89
***p***	< 0.001	-	0.298	-	0.374	-	< 0.001	-	0.002	-

PPV, positive predictive value; NPV, negative predictive value; CI, confidence interval; CE-T1W, contrast-enhanced T1W; CE-T2-FLAIR, contrast-enhanced T2-FLAIR.

*p*-value less than 0.05 is significant.

The inter-reader agreement was almost perfect for both sequences. Cohen’s kappa was higher for CE-T2-FLAIR (κ = 1.0) than CE-T1W (κ = 0.94). Image quality was nearly excellent for both pre-contrast and post-contrast T1W and T2-FLAIR sequences (mean score = 4.83). Diagnostic confidence for meningitis was higher for CE-T2-FLAIR (mean score = 4.83) than CE-T1W (mean score = 4.64).

### Quantitative assessment

**FIGURE 7:**
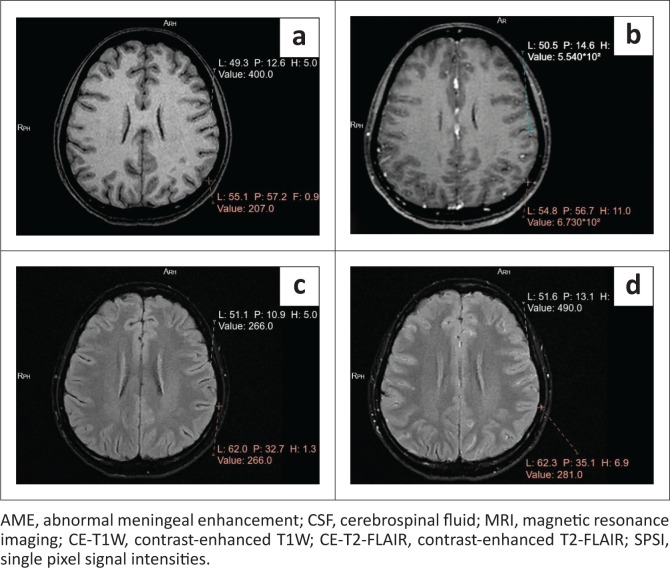
Axial brain MRI of a 22-year-old female patient with CSF proven meningitis showing placement of the region of interest (ROI) and measurement of SPSI. The values in white represent meningeal SPSI and the values in red represent vascular SPSI. (a) Pre-contrast T1W image. (b) CE-T1W image where AME is inconspicuous. Single pixel signal intensities of the meninges (554) are less than the SPSI of the vessels (673). (c) Pre-contrast T2-FLAIR image. (d) CE-T2-FLAIR where AME is clearly visualised. Single pixel signal intensities of the meninges (490) are higher than the SPSI of the vessels (281).

**FIGURE 8:**
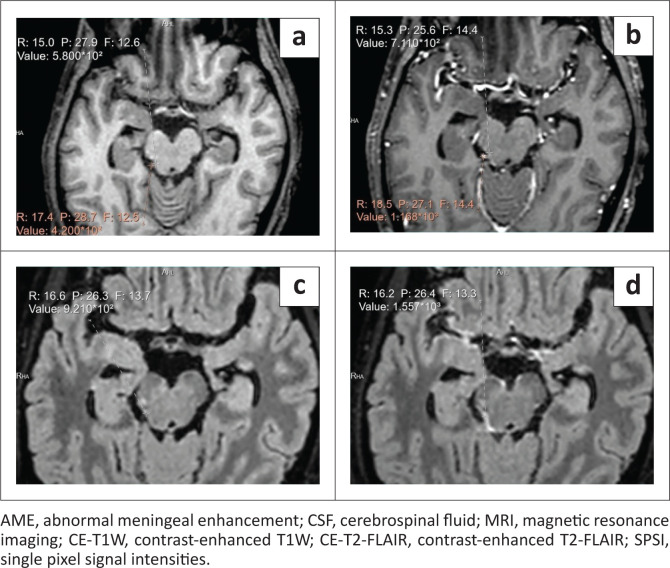
Axial MRI of a 45-year-old male patient with CSF proven meningitis showing placement of the the region of interest (ROI) and measurement of SPSI. The values in white represent meningeal SPSI and the values in red represent vascular SPSI. (a) Pre-contrast T1W image. (b) CE-T1W image where AME is inconspicuous. Single pixel signal intensities of the meninges (711) are less than the SPSI of the adjacent vessels (1168). (c) Pre-contrast T2-FLAIR image. (d) CE-T2-FLAIR image where AME is clearly visualised. Single pixel signal intensities of the meninges are (1557). No adjacent vessel was visualised in the representative axial section.

Quantitative assessment was performed on all patients with meningeal abnormalities by measuring the SPSI of the meninges and vessels. The meningeal and vascular SPSI and enhancement on CE-T1W and CE-T2-FLAIR have been represented in Table 3 (Figure 7 and Figure 8). On CE-T2-FLAIR, all patients displayed a meningeal SPSI and meningeal enhancement higher than vascular SPSI and vascular enhancement, respectively. On CE-T1W, all patients displayed a meningeal SPSI and meningeal enhancement lower than vascular SPSI and vascular enhancement, respectively.

**TABLE 3:** Comparison of contrast-enhanced T1W and contrast-enhanced T2-FLAIR in quantitative assessment of meningitis.

Parameters	Mean	s.d.	Range	Interpretation
**CE-T1W**				
Meningeal SPSI	835.12	181.36	499.0 to 1097.0	On CE-T1W, the meningeal SPSI is significantly lower than vascular SPSI (*p* < 0.01)
Vascular SPSI	1156.26	170.76	937.5 to 1714.0	
Meningeal SPSI – vascular SPSI	−321.14	158.52	−665.5 to -38.0	
Meningeal enhancement	357.08	149.6	80.0 to 679.0	On CE-T1W, the meningeal enhancement is significantly lower than vascular enhancement (*p* < 0.01)
Vascular enhancement	768.9	174.31	461 to 1282.5	
Net enhancement	−411.82	166.31	−833.5 to -132.0	
**CE-T2-FLAIR**				
Meningeal SPSI	1234.46	500.08	566.5 to 2345.0	On CE-T2-FLAIR, the meningeal SPSI is significantly higher than vascular SPSI (*p* < 0.01)
Vascular SPSI	487.64	183.65	240.5 to 1005.5	
Meningeal SPSI – vascular SPSI	746.82	408.89	248.5 to 1693.5	
Meningeal enhancement	781.36	467.52	271 to 1787.5	On CE-T2-FLAIR, the enhancement of meninges is significantly higher than that of vessels (*p* < 0.01)
Vascular enhancement	213.38	156.35	40.0 to 668.0	
Net enhancement	567.98	377.76	191.5 to 1406.5	

Note: Enhancement = post-contrast SPSI – pre-contrast SPSI. Net enhancement = meningeal enhancement – vascular enhancement.

s.d., standard deviation; SPSI, single pixel signal intensity; CE-T1W, contrast-enhanced T1W; CE-T2-FLAIR, contrast-enhanced T2-FLAIR.

The inter-reader agreement on CE-T2-FLAIR was excellent for meningeal SPSI (ICC = 0.98) and vascular SPSI (ICC = 0.97). The inter-reader agreement on CE-T1W was moderate for meningeal SPSI (ICC = 0.69) and good for vascular SPSI (ICC = 0.79).

## Discussion

The current study reiterates that CE-T2-FLAIR is better at detecting AME than CE-T1W. Contrast-enhanced T2-FLAIR had a significantly higher sensitivity, NPV and diagnostic accuracy compared to CE-T1W in the detection of AME in meningitis. Contrast-enhanced T2-FLAIR was able to appreciate more cases of AME than CE-T1W, particularly of subtle grade 1 leptomeningeal enhancement and basal cisternal enhancement around the brainstem. This is because of the suppression of the vascular signal on CE-T2-FLAIR. Subtle meningeal enhancement on CE-T1W may be missed against the higher signal intensity of the vessels in the subarachnoid space. Contrast-enhanced T2-FLAIR showed slightly higher diagnostic confidence and inter-reader agreement than CE-T1W for qualitative assessment. There was a greater SPSI for enhancing meninges compared to vessels on CE-T2-FLAIR, while the SPSI for meninges was significantly lower than vessels on CE-T1W. Inter-reader agreement for quantitative assessment of meningeal enhancement was moderate for CE-T1W and excellent for CE-T2-FLAIR. Thus, it was easier to detect AME on CE-T2-FLAIR on the background of vessel signal suppression, compared to CE-T1W. However, CE-T2-FLAIR may also be associated with false positive meningeal enhancement in the posterior fossa and base of the brain. In this study, the ROI was manually placed for the measurement of SPSI on areas of AME and adjacent vessels. If no patient movement occurs between sequences, software can be used to simultaneously place the ROI at the same site in these sequences. In the study by Azad et al.,^10^ the ROI was placed on the choroid plexus vessels for measurement of the vascular SPSI. Because there may be diagnostic confusion about differentiating vascular enhancement from AME, this study considered vessels adjacent to AME for the measurement of SPSI.

The role of imaging in meningitis is to confirm suspected meningitis, rule out raised intracranial pressure, assess complications and evaluate for a possible aetiology or source of infection.^5^ Imaging with MRI is important for the early detection of meningitis and its complications so that appropriate treatment can be initiated.^8^ Imaging features may include FLAIR sulcal hyperintensity because of increased protein content, AME, enlarged sulcal spaces and complications such as cerebral abscess, diffuse cerebral oedema, subdural effusions, ventriculitis, hydrocephalus, infarcts, vasculitis and cranial nerve involvement.^2,5,9^ Abnormal meningeal enhancement in infectious meningitis is usually leptomeningeal and is because of the breakdown of the blood-brain barrier as a result of inflammatory cytokines or bacterial toxins.^4,5^ Enhancement may also occur as a result of vascular congestion.^6^ Intravenous gadolinium-based MRI contrast agents are administered to identify meningeal or parenchymal enhancement, followed routinely by the T1-weighted (T1W) sequence with or without fat saturation (FS). Enhancement following contrast administration may be intravascular or interstitial. The intact blood-brain barrier in the normal brain, spinal cord, proximal cranial and spinal nerves prevent the leakage of contrast across vessel walls into the interstitium. Any pathological alterations in the permeability of the blood-brain barrier result in interstitial enhancement.^4^

Pia-arachnoid or leptomeningeal enhancement appears as enhancement along the surface of the brain, extending into the subarachnoid space of sulci and cisterns. It usually occurs because of infectious meningitis but can be seen in other conditions such as inflammatory diseases, vasculitis, meningeal carcinomatosis and stroke. Dura-arachnoid or pachymeningeal enhancement, which appears as curvilinear enhancement deep to the inner table of the skull, is rarely observed in infectious meningitis. Instead, it is more commonly associated with other conditions such as intracranial hypotension, granulomatous or inflammatory diseases (e.g., sarcoidosis, infection, IgG4-related pachymeningitis), autoimmune diseases, postoperative changes, meningioma, secondary lymphoma, metastases or it may be idiopathic. Additionally, combined leptomeningeal and pachymeningeal enhancement can also be observed. Other causes of meningeal enhancement include subarachnoid haemorrhage (SAH), drug-induced, vaccines, collagen vascular diseases and chemical meningitis.^2,3,4,9,5,16^

The possible aetiology of AME can be determined based on the type of AME (leptomeningeal or pachymeningeal), grade of AME (higher grade in tubercular, intermediate in bacterial and lower grade in viral aetiology),^10^ morphology of AME (thin, linear in bacterial or viral aetiology; thick, lumpy, nodular in fungal or neoplastic aetiology),^5^ location of AME (cerebral convexity-acute meningitis; basal cisterns- tubercular, chronic meningitis; limbic system-viral),^5,17^ pattern of AME (diffuse or circumscribed enhancement which may involve the infratentorial region occurs in meningitis; focal or multifocal early meningeal enhancement within minutes may occur in ischaemic and haemorrhagic stroke, multiple sclerosis and Susac syndrome; unilateral, thin, leptomeningeal enhancement may be because of non-infectious diseases, such as neurosarcoidosis, vasculitis and Sturge–Weber syndrome) and morphology of associated findings such as parenchymal lesions.^2,9,17^ Parenchymal lesions in infections can be assessed using diffusion-weighted imaging (DWI), susceptibility-weighted imaging (SWI), MR spectroscopy and perfusion imaging, in addition to post-contrast sequences.^5,18^

The use of T1W sequences is associated with excessive vascular enhancement, inflow effects and flat images. The FLAIR sequence allows for the recovery of the brain magnetisation, suppresses CSF signal intensity, helps to reduce CSF artefacts and obtains very high T2 weighting with the use of long echo times. It suppresses the signal from slow-flowing vessels because of the lack of inflow enhancement phenomenon.^6,10^ Thus meningeal enhancement can be more easily detected on CE-T2-FLAIR than on CE-T1W, consistent with many previous studies, some of which have used CE-T1W with fat saturation (FS).^6,8,10,11,12,13^ A study by Galassi et al. in 2002 showed CE-T1W with FS to be better than CE-T2-FLAIR for the detection of meningitis. The discrepancy in their study could be because of the low sample size and lower CSF protein concentration.^13,19^ Contrast-enhanced T1-weighted with FS is not routinely used and was not used in this study.^8,19^ Meningeal enhancement close to fatty marrow in bones may be better visualised with FS than without FS.^19^ Apart from AME, CE-T2-FLAIR can also better visualise parenchymal lesions in infections, demyelinating diseases and metastases.^13,20,21^

Post-processing software can create subtraction images by subtracting the signal intensity of pre-contrast and post-contrast sequences. These subtraction images of pre-contrast and post-contrast FLAIR show higher sensitivity and NPV for detecting leptomeningeal enhancement compared to CE-T1W or CE-T2-FLAIR sequences.^20^ Subtraction images help reduce the scan interpretation time, decrease false positive results and enhance accuracy. However, subtraction techniques may not be possible in all cases because they are susceptible to even minor patient motion artefacts and CSF pulsation artefacts.^21^ Advanced motion autocorrection algorithms may be required to routinely use subtraction techniques.^20^

A new imaging technique for post-contrast study is T1W with MT which uses an off-resonance saturation pulse before the 90° pulse. A study by Azad et al. found that it had lower sensitivity, specificity, PPV, NPV and accuracy than CE-T2-FLAIR.^10^ However, it is useful for the detection of type of meningitis, with tubercular meningitis having a lower MT ratio than bacterial and fungal meningitis and a higher MT ratio than viral meningitis.^10,18^ The 3D T1 sampling perfection with application-optimised contrast using different flip-angle evolutions (T1-SPACE) is a new fast spin echo (FSE) sequence which uses multiple refocussing pulses to generate the MT effect. It has the advantages of a high signal-to-noise ratio, better contrast enhancement, non-visualisation of normal vessels and the ability to detect vessel wall enhancement. A study by Mishra et al. showed that PC-T1-SPACE was not as good as CE-T2-FLAIR for meningeal enhancement overall, except in specific situations such as leptomeningeal enhancement in the < 1-year age group, or associated with gyral or white matter oedema, cranial nerve or ependymal enhancement, in which PC-T1-SPACE was better than CE-T2-FLAIR. The main limitation of PC-T1-SPACE was that it was less effective in the detection of basal cisternal and cerebellar folia enhancement.^11^ Another study by Jeevanandham et al. showed that PC-T1-SPACE was better than CE-T2-FLAIR for meningeal enhancement, except for basal cisternal enhancement.^22^ Apart from T1-SPACE, other contrast-enhanced T1 black blood imaging techniques have also been shown to improve the detection of leptomeningeal enhancement, because of suppression of vascular signal.^23^

The limitations of T2-FLAIR sequence include artefactual hyperintense sulci in children even on pre-contrast FLAIR because of longer effective TE and CSF flow artefacts, artefactual hyperintensity in the posterior fossa, FLAIR hyperintensity in the subarachnoid space in conditions other than meningitis such as SAH, stroke, carcinomatosis, melanosis, fat-containing tumours and in patients who have received supplemental oxygen, gadolinium or iodinated contrast in the previous week or some sedatives.^5,6,9,10^ Other sequences such as SWI (blooming in SAH), DWI (diffusion restriction in stroke), T1W (melanin, fat appear hyperintense) and fat suppression (fat appears hypointense) sequences may be helpful to rule out these non-infectious-conditions-producing FLAIR hyperintensity.^24^ Contrast-enhanced T2-FLAIR is less effective for leptomeningeal enhancement in infants (because of the presence of unmyelinated white matter which is hyperintense) and on the background of gyral or white matter oedema.^11^ The use of the balanced steady state free precession line acquisition with undersampling (BLADE) technique (a Turbo spin echo sequence that uses the PROPELLER [periodically rotated overlapping parallel lines with enhanced reconstruction] k-space trajectory) can help to reduce motion and pulsation artefacts seen in FLAIR.^25^ Cerebrospinal fluid pulsation artefacts can be minimised using techniques like adiabatic inversion pulses, specific radiofrequency pulses, raising the number of interleaving acquisitions and k-space reordering by inversion time at every slice position.^24^

This study has a few limitations such as the lack of correlation with the clinical severity of meningitis (Glasgow Coma Scale scores) and aetiological agent of meningitis, because of the retrospective nature of the study. For acquiring SPSI, the ROI was manually placed in nearly the same position on pre-contrast and post-contrast sequences because there was a slight change in patient position between sequences in a few patients, which was unavoidable in a retrospective study. The subtraction technique could not be used because of minor patient movement between sequences. Patient movements between sequences could be avoided in a prospective study.

This study shows that early meningitis which shows only subtle meningeal enhancement may be detected using CE-T2-FLAIR and missed if only CE-T1W sequence is used. The better diagnostic performance of CE-T2-FLAIR has been quantitatively confirmed in this study. Thus CE-T2-FLAIR should be routinely considered for use in all clinically suspected cases of meningitis. Contrast-enhanced T1-weighted images are routinely acquired immediately after the administration of contrast and can be followed by the CE-T2-FLAIR sequence, which is performed 4 min to 5 min after the start of the CE-T1W sequence, as was performed in this study. However, it must be interpreted in the appropriate clinical context, as non-infectious conditions may also result in AME.

## Conclusion

Contrast-enhanced T2-FLAIR is more effective at detecting AME in meningitis compared to CE-T1W, particularly in cases with subtle enhancement. This is because of the significantly greater signal intensity and enhancement of the meninges relative to the vessels, as quantitatively assessed using SPSI in this study.
